# Barriers to successful care for chronic kidney disease

**DOI:** 10.1186/1471-2369-6-11

**Published:** 2005-10-27

**Authors:** Oliver Lenz, Durga P Mekala, Daniel V Patel, Alessia Fornoni, David Metz, David Roth

**Affiliations:** 1 Division of Nephrology and Hypertension, University of Miami Miller School of Medicine, Miami, FL, USA

## Abstract

**Background:**

The National Kidney Foundation has formulated clinical practice guidelines for patients with chronic kidney disease (K/DOQI). However, little is know about how many patients actually achieve these goals in a dedicated clinic for chronic kidney disease.

**Methods:**

We performed a cross-sectional analysis of 198 patients with an estimated glomerular filtration rate of less than 30 ml/min/1.73 m^2 ^and determined whether K/DOQI goals were met for calcium, phosphate, calcium-phosphate product, parathyroid hormone, albumin, bicarbonate, hemoglobin, lipids, and blood pressure.

**Results:**

We found that only a small number of patients achieved K/DOQI targets. Recent referral to the nephrologist, failure to attend scheduled clinic appointments, African American ethnicity, diabetes, and advanced renal failure were significant predictors of low achievement of K/DOQI goals.

**Conclusion:**

We conclude that raising awareness of chronic kidney disease and K/DOQI goals among primary care providers, early referral to a nephrologist, the exploration of socioeconomic barriers and cultural differences, and both patient and physician education are critical to improve CKD care in patients with Stage 4 and 5 CKD.

## Background

The National Kidney Foundation has recently launched a major effort to define Chronic Kidney Disease (CKD) and formulate clinical practice guidelines [1,2]. It has been clearly shown that complications of CKD, such as anemia, metabolic acidosis, nutritional deficits, secondary hyperparathyroidism, and hypertension, significantly contribute to morbidity and mortality [3-10]. It has been proposed that care for patients with CKD be best delivered in dedicated CKD clinics that provide a multidisciplinary approach to patients with CKD [11,12]. Typically, these clinics are staffed with nephrologists, dieticians, social workers, and educators, and the team works closely with vascular surgeons for access placement. However, little is known about the effectiveness of these clinics at academic centers. The purpose of this cross-sectional analysis is determine to what extent K/DOQI goals are achieved in a dedicated CKD clinic serving a urban, socio-economically disadvantaged minority population.

## Methods

### Patients

**Figure 1:**
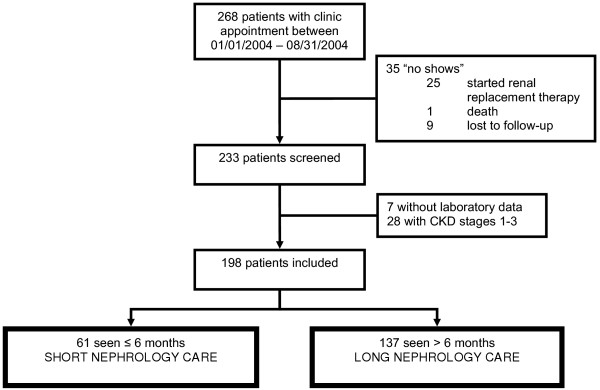
**Recruitment**. 268 patients had an appointment in the CKD clinic. Of those, 233 patients were seen at least once during the study period, and 198 met inclusion criteria.

IRB approval for this study was obtained from the Human Subjects Research Office of the University of Miami, Miami, FL (protocol number 2004–3071). All study procedures were carried out in accordance with the Declaration of Helsinki regarding research involving human subjects. Our chronic kidney disease (CKD) clinic focuses on the care of patients with an estimated GFR, using the modified MDRD formula [1], of less than 30 ml/min/1.73 m^2 ^(Stage 4 and 5). The clinic setting is described in more detail below. We screened 268 patients with an appointment scheduled between January and August of 2004 in the CKD clinic at Jackson Memorial Hospital, Miami, FL. Of those, 35 were excluded because they were not seen: 25 had already started renal replacement therapy, 1 patient had died, and 9 patients were lost to follow-up. Of the remaining 233 patients, an additional 35 were excluded: 7 patients did not have any laboratory data and thus the degree of their renal impairment could not be determined, and 28 patients had CKD Stages 1, 2 or 3 after having recovered from an episode of acute renal failure (Figure 1). No dialysis patients were included in this study. For patients who initiated renal replacement therapy during the study period, their last pre-dialysis parameters were used for analysis.

### Dedicated CKD clinic

All patients with Stages 4 or 5 CKD are followed in our dedicated CKD clinic. The clinic is located in an urban county hospital that predominantly serves socio-economically disadvantaged minority populations. The clinic takes place once a week. The clinic is staffed by two academic nephrologists and four senior nephrology fellows, a nurse practitioner, a social worker, a nurse educator, a case manager, a dietician, and a pharmacist. Patients with stage 4 CKD are seen by a physician at least every 3 months, more frequently if they have laboratory abnormalities or uncontrolled blood pressure. Patients with stage 5 CKD are seen monthly. Patients receiving recombinant erythropoietin for anemia or active vitamin D for secondary hyperparathyroidism are seen monthly by the nurse practitioner. The nurse educator gives a short lecture about dialysis options, vascular access, and complications of CKD at the beginning of each clinic session. In addition, one-on-one educational sessions are offered to patients who decide on a dialysis modality. The dietician accepts walk-in appointments during regular CKD clinic hours in addition to elective appointments for nutritional counseling. The case manager assists with referrals and follow-up appointments. The social worker assists mainly with financial issues, since most of our patients belong to socio-economically disadvantaged minority populations, as well as referrals to the transplant center. The pharmacist is available to answer patients' questions about their medications, drug and food interactions, adverse events, as well as programs offering financial assistance.

Nephrology fellows act as the patients' primary nephrologist. Each case is discussed with a staff nephrologist. Following a checklist, current data for core indicators for CKD care including anemia, calcium, phosphate and PTH metabolism, nutrition, metabolic acidosis, hyperlipidemia, blood pressure, and dialysis options including vascular access, are compared to target values. Algorithms are in place to start and adjust phosphate binders, calcium supplements, active vitamin D preparations, recombinant erythropoietin, iron supplementations, and to make decision regarding vascular access placement and transplant referral. These algorithms are based on published treatment guidelines [2,13-15]. K/DOQI guidelines do not contain treatment recommendations for low HDL, which we commonly treat with extended-release niacin.

Laboratory data are obtained quarterly for patients with stage 4 CKD and monthly for patients with stage 5 CKD. In patients not at goal, and in those receiving active vitamin D preparations or recombinant human erythropoietin supplementation, data are obtained monthly, and medication doses are adjusted accordingly.

The nephrology clinics receive referrals mostly from primary care providers (PCP), internists, cardiologists, and endocrinologists practicing within the hospital system and its affiliated community clinics. All referrals are triaged by their estimated GFR: patients with stage 4 or 5 CKD are directly seen in the CKD clinic, while all others are seen in the general nephrology clinic. PCP are strongly encouraged to refer all patients with Stage 3 CKD, or whenever there is a doubt in regards to diagnosis or therapy. During the study period, of the patients seen for the first time in the CKD clinic about half came from the general nephrology clinic with progressive renal failure, while the other half were new referrals. In patients seen in the nephrology clinics who reach Stage 4 CKD the nephrologist typically manages all problems related to chronic kidney disease.

### Data collection

Laboratory parameters for serum calcium, phosphate, intact PTH, albumin, bicarbonate, and hemoglobin were obtained from chart review. For each patient, the most recent value prior to their last clinic appointment within the study period was used. Laboratory data had to be obtained no more than 3 months prior to the patients scheduled visit, otherwise they were entered as "not at goal". This is based on the K/DOQI recommendation to obtain laboratory data for the parameters investigated in this study at least quarterly. The serum calcium concentration was corrected for serum albumin using the formula [Ca_corrected_] = [Ca_measured_] + 0.8 × (4 - [Albumin]), where [Ca] is expressed in mg/dl and [Albumin] is expressed in g/dl [16]. The calcium-phosphate product was obtained by multiplying the corrected serum calcium with the serum phosphate concentration and expressed as mg^2^/dl^2^. The following treatment targets were used for patients with CKD Stages 4 and 5, respectively [2,13-15]: calcium 8.4–10.3/8.4–9.5 mg/dl, phosphate 2.7–4.6/3.5–5.5 mg/dl, and intact PTH 70–110/150–300 pg/ml. Identical treatment targets were set for patients with CKD Stage 4 and 5, respectively, for calcium-phosphate product (<55 mg^2^/dl^2^), albumin (≥3.5 g/dl), bicarbonate (>22 mmol/l), hemoglobin (≥ 11 g/dl), and blood pressure (less than 130/80 mmHg). The goals for lipid control were set at LDL < 100 mg/dl, triglycerides < 500 mg/dl, and non-HDL < 150 mg/dl. If any of the three lipid goals was not met, the subject was classified as "not at goal". Thus, a total of nine parameters were evaluated: 8 laboratory parameters plus blood pressure. Patient age, gender, ethnicity, and the following co-morbidities were abstracted from chart review: diabetes, hypertension, and hyperlipidemia. Failure to attend scheduled clinic visits half the time or more (no show rate ≥ 50%) was used as a surrogate measure of non-adherence. The length of nephrology care in months was tabulated; time spent in the CKD clinic, the general nephrology clinic, or under the care of a nephrologist in private practice was added. Patients who had been seen for 6 months or less by a nephrologist were categorized as having received short nephrology care (SNC), while all others were categorized having received long nephrology care (LNC).

### Statistical analysis

All statistical analyses ware carried out using the SPSS statistical software package (SPSS Inc., Chicago, IL). We chose poor achievement of K/DOQI goals as the outcome variable, which was defined as having less than half the parameters, i.e., 0 to 4 out of 9, at goal, because we felt that it is clinically relevant if a patient has more than half of the investigated parameters not at goal. Categorical variables were compared by chi square analysis. Continuous variables were compared with Student's t-test if two groups were present or ANOVA followed by post hoc analysis if more than two groups were present. Welch's correction for unequal variances and Bonferroni's correction for multiple testing were employed where indicated. Associations were tested by logistic regression. The following variables were thought to be clinically relevant and entered into the model: age (converted into decades), gender, African American ethnicity, short nephrology care (SNC), failure to attend scheduled clinic visits (no show rate above 50%), stage 5 CKD, diagnosis of diabetes, hypertension, and hyperlipidemia.

## Results

### Patient characteristics

Patient characteristics are shown in Table 1. Median follow-up time for SNC patients was 2 months, while the median follow-up for LNC patients with nephrology care was 33 months. 44% of LNC patients had been followed for 3 years or more. Important differences between LNC patients and SNC were noted. There were fewer African Americans and more young patients among SNC patients. SNC patients were less likely to carry the diagnosis of hypertension and hyperlipidemia but more likely to have diabetes. About 43% of the patients were poor and uninsured, 18% had Medicaid only, and 26% had Medicare as their primary insurance. There was no difference in insurance status between SNC or LNC patients (data not shown).

**Table 1:** Patient characteristics

	All (N = 198)	LNC^† ^(N = 146)	SNC^† ^(N = 52)	P-value
**Ethnicities**				
Hispanic	46%	44%	54%	0.214
African American	43%	48%	31%	0.032
Haitian	7%	7%	8%	0.839
Caucasian	3%	1%	5%	0.083
Asian	1%	0%	2%	0.093

**Age [Years]**				
Mean ± SD	56 ± 13.4	51 ± 14	58 ± 12.7	0.001
<31	4%	1%	10%	0.006
31–40	10%	8%	14%	0.270
41–50	20%	19%	21%	0.758
51–60	25%	23%	31%	0.241
61–70	28%	32%	17%	0.041
71–80	12%	13%	8%	0.304
>80	3%	3%	2%	0.176

**Gender**				
Female	53%	55%	48%	0.405

**CKD Stage**				
Stage 4	47%	48%	44%	0.645

**Length of Nephrology Care [Months]**				
0–1	9%	0%	35%	-
2–6	17%	0%	65%	-
7–12	14%	20%	0%	-
13–24	15%	22%	0%	-
24–36	15%	21%	0%	-
>36	30%	44%	0%	-

**Co-Morbidities**				
Hypertension	92%	95%	83%	0.004
Hyperlipidemia	66%	71%	52%	0.015
Diabetes	50%	45%	65%	0.008

**Estimated GFR at Referral to Nephrology [ml/min/1.73 m^2^]**				
Mean ± SD	26 ± 14.9	29 ± 15.7	17 ± 8.3	<0.001
<15	21%	13%	42%	<0.001
15–29	52%	51%	52%	0.945
30–59	24%	31%	6%	<0.001
>59	4%	5%	0%	0.108

^† ^LNC: seen by a nephrologist for more than 6 months; SNC: seen by a nephrologist for 6 months or less. Data for LNC and SNC were compared using Chi Square Analysis for categorical variables or by unpaired t-test for continuous variables. Welch's correction was used if unequal variances were present.

### Achievement of K/DOQI goals

The median values along with the 25^th ^and 75^th ^percentiles for estimated GFR, serum calcium, phosphate, calcium-phosphate product, intact PTH, bicarbonate, albumin, hemoglobin, lipids, and blood pressure are shown in Table 2. The proportion of patients achieving K/DOQI goals are shown in Table 3.

**Table 2:** Mean laboratory parameters and blood pressure values

	Stage 4 CKD		Stage 5 CKD		
	SNC^† ^(N = 23)	LNC^† ^(N = 70)	SNC^† ^(N = 29)	LNC^† ^(N = 76)	p-values
Estimated GFR [ml/min/1.73 m^2^]	22 ± 4*	21 ± 4^#^	9.1 ± 3*	9.1 ± 3^#^	*^#^<0.001
Calcium [mg/dl]	9.5 ± 0.6	9.3 ± 0.6*	8.9 ± 0.9	8.9 ± 0.8*	*0.015
Phosphate [mg/dl]	4.6 ± 1.1	4.1 ± 0.9*	5.2 ± 1.2	5.3 ± 1.5*	*<0.001
Calcium-Phosphate Product [mg^2^/dl^2^]	45 ± 11	38 ± 9*^#^	46 ± 10^#^	47 ± 13*	*<0.001; ^#^0.035
iPTH [pg/ml]	155 ± 153*	203 ± 152^#^	532 ± 476*	486 ± 385^#^	*0.008; ^#^<0.001
Bicarbonate [mmol/l]	23 ± 4	25 ± 4*^#^	21 ± 5^#^	21 ± 4*	*^#^<0.001
Albumin [g/dl]	3.3 ± 0.8	3.8 ± 0.5	3.5 ± 0.7	3.6 ± 0.6	
Hemoglobin [g/dl]	11.3 ± 2.0	11.8 ± 1.2*^#^	10.3 ± 1.9^#^	10.5 ± 1.8*	*<0.001; ^#^0.003
Total Cholesterol [mg/dl]	190 ± 37	189 ± 40	180 ± 56	172 ± 41	
Triglycerides [mg/dl]	153 ± 72	176 ± 120	183 ± 115	149 ± 85	
LDL [mg/dl]	111 ± 30	103 ± 34	100 ± 49	94 ± 33	
HDL [mg/dl]	48 ± 16	53 ± 15*	43 ± 15*	47 ± 16	*0.033
Non-HDL [mg/dl]	142 ± 38	137 ± 39	137 ± 54	125 ± 37	
Systolic Blood Pressure [mmHg]	148 ± 27	135 ± 26*	155 ± 27*	147 ± 28	
Diastolic Blood Pressure [mmHg]	82 ± 21	73 ± 14*	84 ± 17*	77 ± 16	*0.049

Shown are the most recent data obtained within the 3-months period preceding the last clinic visit. All data are shown as mean ± standard deviation. Stage 4 = GFR 15–29, Stage 5 = GFR <15 ml/min/1.73 m^2^. The glomerular filtration rate was estimated using the modified MDRD formula [1]. ^† ^SNC: nephrology care for 6 months or less; LNC: nephrology care for more than 6 months. Data in each row were analyzed by one-way ANOVA followed by pair wise post hoc comparisons. Welch's correction for unequal variances was used where indicated. Bonferroni's correction for multiple comparisons was applied within each row. Pairs compared in each row bear the same symbol. Only p < 0.05 are shown.

**Table 3:** Proportion of patients achieving K/DOQI targets

	Stage 4 CKD			Stage 5 CKD		
	SNC^† ^(N = 23)	LNC^† ^(N = 70)	p-value	SNC^† ^(N = 29)	LNC^† ^(N = 76)	p-value
Calcium	87%	91%	0.529	79%	82%	0.791
Phosphate	48%	80%	0.003	52%	66%	0.185
Calcium-Phosphate Product	65%	94%	<0.001	83%	78%	0.789
iPTH	9%	20%	0.213	24%	21%	0.733
Bicarbonate	70%	81%	0.230	38%	49%	0.323
Albumin	43%	76%	0.004	48%	62%	0.208
Hemoglobin	57%	77%	0.056	38%	42%	0.697
Lipids	35%	49%	0.249	34%	49%	0.191
Triglycerides	91%	94%	0.614	90%	96%	0.207
LDL	39%	53%	0.253	48%	55%	0.521
Non-HDL	52%	63%	0.253	66%	76%	0.249
Blood Pressure	17%	34%	0.132	10%	17%	0.382

Shown are the percentages of patients in each group with laboratory values within K/DOQI target ranges. Laboratory data were the most recent values within the 3 months period preceding the last clinic visit. See methods section for individual ranges. Stage 4 = GFR 15–29, Stage 5 = GFR <15 ml/min/1.73 m^2^. The glomerular filtration rate was estimated using the modified MDRD formula [1]. ^† ^LNC: seen by a nephrologist for more than 6 months; SNC: seen by a nephrologist for 6 months or less. Data for LNC and SNC were compared using Chi Square Analysis. Only p-values < 0.05 are shown.

Except for SNC patients with Stage 5 CKD more than 80% of all patients achieved calcium goals. Those outside the target range were more likely to have low calcium levels suggesting secondary hyperparathyroidism; calcium levels above target range were rare.

80% of LNC patients with Stage 4 CKD had phosphate levels at goal, and only 1 patient had a serum phosphate above 6 mg/dl. Only 66% of LNC patients with Stage 5 CKD had phosphate levels within the target range, 22% had phosphate levels above 6 mg/dl, and 13% had phosphate levels above 7 mg/dl. The highest phosphate level in this group was 10.5 mg/dl. Among SNC patients, only about half of all patients achieved phosphate goals, and 13% with Stage 4 CKD and 24% with Stage 5 CKD had phosphate levels above 6 mg/dl. None of these patients had serum phosphate levels above 7 mg/dl, suggesting a shorter disease course or poorer nutrition.

Given the relative high percentage of patients with low calcium concentrations, calcium-phosphate products were within target range for more than three quarters of all patients. The lower percentage of SNC patients with Stage 4 CKD achieving goal is due to the high number of missing data that were coded as not at goal. Missing data resulted from missing phosphate determinations.

Secondary hyperparathyroidism presented a serious problem in our cohort. Only between 9% and 24% of all patients achieved PTH goals. Among those with Stage 4 CKD who had a PTH determination on file, PTH levels were below target in 6%, 41% were at goal, 110–200 pg/ml in 24%, 200–400 pg/ml in 24%, and above 400 pg/ml in 5% of the patients. None of the patients with Stage 4 CKD had PTH levels above 1000 pg/ml. For those with Stage 5 CKD who had a PTH determination on file, PTH levels were below target in 15%, at goal in 29%, 300–600 pg/dl in 28%, 600–900 pg/ml in 17% and above 900 pg/ml in 11% of the patients. Particularly in SNC patients, missing PTH determinations that were coded as not at goal resulted in the overall very low number of patients achieving PTH targets.

Between 70% and 80% of patients with Stage 4 CKD had good control of metabolic acidosis, while only 38% to 49% of patients with Stage 5 CKD had adequate bicarbonate levels. There was no significant difference between SNC and LNC patients.

Albumin was chosen as a surrogate marker for malnutrition. A higher proportion of LNC patients had normal serum albumin concentrations than SNC patients; however, this difference was no longer significant in patients with Stage 5 CKD. 9% of LNC patients with Stage 4 CKD and 13% of LNC patients with Stage 5 CKD had albumin concentrations below 3 g/dl. For SNC patients, the corresponding proportions were 35% and 24%, respectively.

More than three quarters of LNC patients with Stage 4 CKD had adequate anemia management, while only 57% of SNC patients with Stage 4 CKD had desirable hemoglobin concentrations. Hemoglobin concentrations for LNC patients with Stage 4 CKD were between 9 and 10 g/dl in 6%, and between 10 and 11 g/dl in 17% of the cases, while for SNC patients they were below 9 g/dl in 9%, between 9 and 10 g/dl in 17%, and between 10 and 11 g/dl in 17% of the cases. Anemia control was worse in patients with stage 5 CKD. For SNC patients and LNC patients hemoglobin concentrations were below 9 g/dl in 21% and 16%, between 9 and 10 g/dl in 28% and 17%, and between 10 and 11 g/dl in 14% and 24% of the cases, respectively.

Hypertriglyceridemia was uncommon in all groups. While only about half of all patients achieved an LDL of less than 100 mg/dl, 64% had an LDL less than 110 mg/dl and 79% had an LDL of less than 130 mg/dl. Total cholesterol levels tended to be lower in patients with Stage 5 CKD, leading to a greater proportion of patients with non-HDL cholesterol at goal.

Blood pressure control was poor in all groups. Stage 1 hypertension [17] was present in 27% and 37% of LNC patients with Stages 4 and 5 CKD, respectively, and in 30% and 52% of SNC patients with Stages 4 and 5 CKD, respectively. Stage 2 hypertension [17] was present in 14% and 28% of LNC patients with Stages 4 and 5 CKD, respectively, and in 35% and 34% of SNC patients with Stages 4 and 5 CKD, respectively. Overall, the prevalence of stage 1 or stage 2 hypertension combined was 82% in SNC and 44% in LNC patients (p < 0.003)

Table 4 shows the percentage of patients with 0–1, 2–3, 4–5, 6–7, and 8–9 of the nine investigated parameters at goal. As became already evident in table 3, our results indicate that LNC patients with Stage 4 CKD who had been seen by a nephrologist for more than 6 months were more likely to achieve goals than SNC patients seen for 6 months or less. In Stage 5 CKD patients, length of nephrology care did not appear to make a big difference. Overall, only a very small fraction of all patients had eight or nine parameters at goal.

**Table 4:** Proportion of patients achieving a set number of K/DOQI targets

	Stage 4 CKD^#^			Stage 5 CKD^#^		
Parameters at goal*	SNC^† ^(N = 23)	LNC^† ^(N = 70)	p-value	SNC^† ^(N = 29)	LNC^† ^(N = 76)	p-value
0–1	0%	0%		0%	5%	
2–3	39%	3%		34%	28%	
4–5	26%	30%		52%	26%	
6–7	35%	54%		14%	36%	
8–9	0%	13%		0%	5%	

Mean ± SD	4.3 ± 1.8	6.0 ± 1.3	0.001	4.1 ± 1.4	4.6 ± 2.0	0.477

* Number of parameters shown in table 3 that are within the range recommended by K/DOQI. See methods section for individual ranges. Each row shows the proportion of patients within each category achieving the indicated number of parameters at goal. The last row shows the mean number of parameters at goal within each category. P-values were determined by ANOVA with Welch's correction for unequal variances followed by Tamhane's post hoc pairwise comparison of SNC and LNC within each Stage of CKD. ^# ^Stage 4 = GFR 15–29, Stage 5 = GFR <15 ml/min/1.73 m^2^. The glomerular filtration rate was estimated using the modified MDRD formula [1]. ^† ^SNC: seen for 6 months or less, LNC: seen for more than 6 months

### Predictors of low achievement of K/DOQI goals

**Figure 2:**
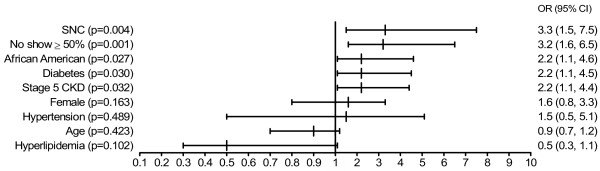
**Logistic regression analysis to identify predictors of poor achievement of K/DOQI Goals**. The outcome was defined as having less than five of the nine parameters shown in Table 3 at goal. Shown are the odds ratio (OR), 95% confidence interval (CI), and p-value. All 2-way interaction terms were tested and no interaction was detected. Thus, interaction terms were excluded from the full model. All variables entered are shown. Short nephrology care (SNC) was defined as having been seen by a nephrologist for 6 months or less. African Americans were compared to all other ethnicities. No show ≥ 50% indicates failure to attend scheduled clinic appointments at least half the time. Age was entered after transformation into decades.

We used logistic regression to determine predictors for the failure to achieve K/DOQI goals (Figure 2). The following variables were entered into the model: failure to attend scheduled clinic appointments (no show rate ≥50% versus <50%), having received short nephrology care (≤6 months versus >6 months), CKD Stage 5 (versus CKD Stage 4), African American ethnicity (versus all others), age below 60 years (versus ≥60), female gender (versus male), and having (versus not having) diabetes, hypertension or hyperlipidemia. In addition, all two-way interaction terms were considered; however, no interactions were detected. Short nephrology care (OR = 3.3), failure to attend scheduled clinic appointments (OR = 3.2), African American background (OR = 2.2), diabetes (OR = 2.2), and Stage 5 CKD (OR = 2.2) were significant predictors of low achievement of K/DOQI goals. Age, gender, hyperlipidemia, and hypertension did not reach the level of significance set as p < 0.05.

## Discussion

Dedicated CKD clinics have been established based on the conviction that such clinics will help implement K/DOQI goals and thus improve outcomes [11,18]. Recent data show that multidisciplinary pre-dialysis care is associated with significantly lower mortality after starting dialysis [19]. However, in this study, *Curtis et al *also report that anemia management only achieved a mean hemoglobin of 102 ± 18 g/l in the multidisciplinary group at the time dialysis was initiated. This is comparable to our results achieved in patients with stage 5 CKD (median: 10.5 mg/dl) and falls short of K/DOQI goals. Similar to our findings, preliminary data from the United Kingdom showed difficulties in achieving K/DOQI goals [20]: in patients with stage 4 and stage 5 CKD, blood pressure goals were achieved in 22.2% and 16.9%, and bicarbonate goals in 41.2% and 29.7%, respectively. Achievement of PTH and hemoglobin goals was better in that study, however, cut-off values were different from the ones recommended by K/DOQI, making a direct comparison difficult. Thus, it appears that although there are proven benefits of having CKD clinics, outcomes might be improved even further if a better achievement of K/DOQI goals could be realized. We found that several factors can be identified that contribute to our failure to reach K/DOQI targets in a larger number of patients.

For most of the parameters investigated, SNC patients with Stage 4 CKD were less likely to achieve goals than LNC patients. Since our referral sources are PCP working either out of the main campus or affiliated satellite clinics, our findings raise the hypothesis that PCP may not be familiar with CKD and its associated co-morbidities. This is most evident in the finding that more than half the patients referred from PCP did not have their PTH level checked, and a quarter had no phosphate determinations on record. The observation that about 25% of the cohort in this study represented SNC patients, i.e., patients referred to the nephrologist for the first time when they already had Stage 4 or 5 CKD, corroborates this impression. The prevalence of CKD is high and with the rising incidence of diabetes it is not expected to decrease [21]. Thus, it will be of vital importance to include the PCP in the care of CKD patients and promote early screening, early initiation of treatment, and timely nephrology referral [22,23]. Educating PCP about early CKD care will be an important step in including them in the care of CKD patients.

We found that the most difficult to manage parameter was PTH; no more than 25% of the patients achieved K/DOQI targets. Similar results were reported by others in dialysis patients despite extensive use of active Vitamin D preparations [24]. A review of our patients' charts revealed that virtually every patient with an elevated PTH was prescribed active Vitamin D, with the exception of those with elevated phosphate levels (above 5.5 mg/dl) or calcium-phosphate products (above 55 mg^2^/dl^2^). However, Vitamin D doses used were quite low compared to what is customary in hemodialysis patients (data not shown). Thus, the problems may not to be lack of use, but failure to utilize the correct dose of active Vitamin D preparations, even though an algorithm was in place. The preferred active vitamin D preparation at this institution was oral doxercalciferol. Based on the prevalence and severity of observed hyperparathyroidism in our clinic and the dosage algorithm used, we expected an average dose of 1.49 micrograms daily in patients with stage 4 CKD and 1.75 micrograms daily in patients with stage 5 CKD. The observed dose in patients that did receive active vitamin D was about 25% lower than the expected dose. "Clinical inertia" on the part of clinicians, which has been reported in the care of patients with other chronic diseases such as diabetes, may represent a significant problem [25], as well as concerns about adynamic bone disease, even though this condition is uncommon in the pre-dialysis population [26-29]. In addition, patients referred late by the PCP may already have hyperplasia of the hyperparathyroid gland that is more difficult to treat than the hypertrophy seen earlier in the course of the disease. Finally, we noticed that a number of patients did not understand the difference between over-the-counter vitamin preparations and active Vitamin D. Taken together these data suggest that both physician and patient education are critical components of CKD care. In light of a recent report showing that the combination of high PTH and normal serum calcium and phosphate concentrations was associated with the lowest mortality in prevalent hemodialysis patients [6], prospective studies correlating PTH levels with outcomes in pre-dialysis patients are needed.

Patients with Stage 5 CKD were less likely to achieve K/DOQI targets when compared to patients with Stage 4 CKD. In fact, the benefit of LNC was virtually lost among patients with stage 5 CKD. Among those with Stage 5 CKD, patients requiring renal replacement therapy had the worst results (data not shown). One might argue that the renal team initiates renal replacement therapy too late, i.e., dialysis is only started at a point when medical management becomes impossible. We noted that a number of patients in our clinic are very reluctant to initiate renal replacement therapy in the absence of severe uremic symptoms despite intense counseling. In fact, only half the patients with a GFR of less than 10 ml/min/1.73 m^2 ^started renal replacement therapy. There are no strong data to suggest that an earlier start of dialysis is beneficial [30-33], however, there is data to suggest that treatment goals for complications of CKD such as anemia are more frequently achieved after initiation of hemodialysis [21]. It is possible that this is secondary better adherence since many medications, such as erythropoiesis-stimulating agents, iron, or active vitamin D preparations, are administered in intravenous form during the hemodialysis session. In addition, regular, mandatory clinical quality assurance measures in dialysis units may lead to a stricter adherence to treatment protocols. Thus, in the CKD population, additional vigilance may be needed in patients approaching the need for renal replacement therapy to achieve better outcomes.

Our finding that failure to attend scheduled clinic appointments, younger age, and being African American were all associated with the failure to achieve K/DOQI goals may suggest that cultural or socioeconomic barriers exist. Prior studies investigating access to nephrology care have reported similar findings [34,35]. It is interesting to note that a large proportion of Hispanic patients have a poor command of English, yet being African American conferred a higher risk of not achieving K/DOQI goals, suggesting that language *per se *may not be a barrier for successful CKD care.

Hyperlipidemia was associated with a better achievement of K/DOQI goals, although statistical significance was not reached. This was surprising since hyperlipidemia has been associated with higher cardiovascular morbidity and mortality, and has been postulated to promote faster progression of renal failure. For this reason, we expected the opposite association. However, since virtually all patients in our cohort carrying the diagnosis of hyperlipidemia are treated with a HMG-CoA reductase inhibitor, we hypothesize that it may be the treatment for, rather than the diagnosis of hyperlipidemia that promotes achievement of K/DOQI goals. Additional research is needed to explore this possibility.

A significant number of variables associated with failure to achieve K/DOQI goals were patient characteristics, such as failure to attend clinic appointments, younger age, and African American ethnicity. This is an important observation, because physician profiling is gaining momentum as a way to improve quality of patient care and adherence to practice guidelines [36]. Our data show that it may be very difficult to judge physicians' performances based on report cards derived from a set of laboratory data that are compared to published practice guidelines, without taking into account the characteristics of the patient population.

### Limitations of the study

Our study has several important limitations. Given that this is a single center study, our population has a unique ethnic mix, which may make it difficult to compare our results with what is seen at other centers. However, minorities are among the fastest growing segments in the United States population, and minorities have the highest incidence and prevalence of chronic kidney disease and end-stage renal disease [37]. Thus, it is of vital importance to focus on the health needs of these populations. The population size is small in comparison to some of the published data from managed care providers [38]. This may decrease our power to detect parameters associated with a poorer outcome. However, our study identified important predictors of poor outcomes that can be targeted in future prospective studies. We chose the most recent laboratory value rather than the mean of a number of tests. Thus, patients who had a recent worsening of their status, for example due to hospitalization, may inadvertently have been classified as 'not at goal'. However, averaging several values may have introduced a bias as well, since SNC patients are expected to have a trend towards improvement. Being a retrospective analysis, our data only show associations and do not reveal causalities. Thus, the hypotheses generated by this study will need to be tested prospectively. We have initiated a prospective clinical study to address several of the key points raised by the current work, such as raising the awareness of CKD among referring physicians, frequent quality checks and direct feed back to providers to avoid "clinical inertia", a modified teaching program for patients to improve the patient-provider interaction and enhance empowerment, and intensified case management to address socio-economic barriers to CKD care. It is not known, and this study was not designed to test, whether achieving K/DOQI goals will improve or potentially worsen long-term-outcomes in patients with Stages 4 and 5 CKD, such as cardiovascular morbidity and mortality. Additional research is needed to address this important question. Finally, it is likely that the parameters collected from a given patient are not independent. In addition, multiple interactions exist; for example, the treatment of hyperphosphatemia may influence metabolic acidosis, the treatment of anemia may affect hypertension, and well-nourished patients may have a higher chance to develop hyperphosphatemia. Given the small sample size in our study, we were not able to identify predictors of poor outcomes for each category, such as anemia, while adjusting for all other terms. While it would be clinically important to test these scenarios, this likely will require a multi-center effort.

## Conclusion

In summary, we found that K/DOQI goals are achieved in only a small proportion of patients cared for in a dedicated CKD clinic. While numerous publications show that dedicated CKD clinics lead to better outcomes, it appears that there is room for improvement. Raising awareness of CKD and K/DOQI goals among PCP, early referral to a nephrologist, timely initiation of renal replacement therapy, the exploration of socioeconomic barriers and cultural differences, and both continuous patient and physician education are critical to improve CKD care. Prospective clinical trials are needed to explore the impact of these measures on cardiovascular morbidity and mortality in the pre-dialysis arena.

## Competing interests

Oliver Lenz has acted as a consultant for Abbott Laboratories, Inc.

## Authors' contributions

Oliver Lenz designed the study, obtained IRB approval, collected most data, performed all statistical analyses, and wrote the manuscript. Durga P. Mekala, Daniel V. Patel, Alessia Fornoni, David Metz, and David Roth were major collaborators in study design, data collection, and manuscript preparation. All authors approved the final version of this manuscript.

## Pre-publication history

The pre-publication history for this paper can be accessed here:


